# Microleakage and Marginal Integrity of Ormocer/Methacrylate-Based Bulk-Fill Resin Restorations in MOD Cavities: SEM and Stereomicroscopic Evaluation

**DOI:** 10.3390/polym15071716

**Published:** 2023-03-30

**Authors:** Aslı A. Şenol, Büşra Karabulut Gençer, Bilge Tarçın, Erkut Kahramanoğlu, Pınar Yılmaz Atalı

**Affiliations:** 1 Department of Restorative Dentistry, Faculty of Dentistry, Marmara University, Istanbul 34854, Turkey; 2 Department of Restorative Dentistry, Faculty of Dentistry, Nişantaşı University, Istanbul 34398, Turkey; 3 Department of Prosthodontic Dentistry, Faculty of Dentistry, Marmara University, Istanbul 34854, Turkey

**Keywords:** bulk-fill composite, layering thicknesses, marginal relocation, microleakage, ormocer

## Abstract

This in vitro study aimed to compare the microleakage and marginal integrity of methacrylate/ormocer-based bulk-fill composite (BFC) restorations used in cervical marginal relocation with two different layering thicknesses in mesio-occlusal-distal (MOD) cavities exposed to thermo-mechanical loading. Standard MOD cavities were prepared in 60 mandibular molars and assigned into three groups: x-tra fil/AF + x-tra base/XB, Tetric N-Ceram Bulk Fill/TNB + Tetric N-Flow Bulk Fill/TFB, and Admira Fusion x-tra/AFX + Admira Fusion x-base/AFB. Each group was further divided into two subgroups (2 mm and 4 mm) based on the thickness of flowable BFCs (*n* = 10). The specimens were subjected to thermo-mechanical loading (240,000 cycles) and immersed in 0.2% methylene blue. Following mesiodistal sectioning, the specimens were examined under stereomicroscope (×25) and scored (0–3) for microleakage. Marginal integrity was examined using a scanning electron microscope (SEM). Descriptive statistical methods and the chi-square test were used to evaluate the data (*p* < 0.05). While there was no statistically significant difference in gingival cement microleakage in the XB and AFB specimens with a 4 mm thickness, microleakage was significantly increased in the TFB specimen (*p* = 0.604, 0.481, 0.018 respectively). A significantly higher amount of score 0 coronal microleakage was detected in the AFX2 mm + AFB4 mm compared to the TNB2 mm + TFB4 mm (*p* = 0.039). The SEM examination demonstrated better marginal integrity in groups with 2 mm thick flowable BFCs. Ormocer and methacrylate-based materials can be used in marginal relocation with thin layers.

## 1. Introduction

Since their development, the advances in composite materials have not only expanded the number of indications for their use but have also improved clinical outcomes in terms of function and aesthetics [1]. Despite these developments, secondary caries remain key limitations that considerably affect the clinical longevity and success of restorations, although their relationship to microleakage and polymerization shrinkage has not yet been clearly demonstrated [2].

The occurrence of microleakage may be indirectly associated with polymerization shrinkage or the development of associated stresses as a result of the exposure of the restoration to thermal, mechanical, and chemical stimuli in the oral environment [3,4]. Recent modifications, such as the inorganic–organic matrix and application techniques in resin-based composites, have attempted to minimize polymerization shrinkage and wear and provide improved marginal integrity and biocompatibility [5].

The incremental technique, which involves the application and polymerization of resin-based composites with layers of a thickness of 2 mm or less, is a common approach that is also involved in the instructions of the manufacturers. The incremental application of composite resin during restoration may help to minimize the formation of gaps, increase light transmittance, decrease the ratio of bonded to unbonded surface, and ensure adequate bonding between the dental tissue and restoration [6]. However, keen attention to details such as isolation, exposure to light, and working time when using the incremental technique for restoration with traditional composite materials is essential. This technique also has several limitations, including an increased seating time and the formation of gaps between the layers [7,8]. The initiation systems and translucency properties of bulk-fill composites (BFC) promise an increased depth of cure. Extended polymerization durations are more likely to lead to the critical temperature values for pulp damage than short polymerization durations, suggesting that the use of bulk-fill composites in the restoration of deep Class II cavities is advantageous to preserve pulp health [9]. Stress-reliever technologies result in the reduction of polymerization shrinkage stresses. BFCs have attempted to address these limitations by increasing the polymerization depth, achieving a better conversion degree by reducing the oxygen inhibition layer and minimizing the risk of the formation of gaps between the layers and subsequent contamination [10,11,12]. However, some studies have resulted in higher shrinkage vector values, a higher debonding tendency [13], and a decrease in hardness with an increasing depth of the bulk application method [14,15]. The light penetration and degree of conversion to be obtained can be questioned due to the increased material thickness in the bulk technique, which includes composite applications with a thickness of 4 mm or greater. In the study conducted by Par et al. [16], it was observed that the material thickness was the most effective factor for polymerization to be completed.

Margin relocation is a current and conservative approach in proximal spaces for which the cervical border extends below the cemento-enamel junction (CEJ). Inadequate adhesion, limited light access, and the difficulties that can be experienced during application in deep Class II cavities devoid of enamel should be compensated for by the increased adaptability and curing depth of the material used [17,18,19]. The use of a low-viscosity composite as the marginal relocation material can ensure superior marginal adaptation with less gap formation as a stress absorbing layer and reduce polymerization shrinkage at the tooth restoration interface [20].

Dental restorative materials using ormocer (Organic-Modification-Ceramic) technology, produced as a result of inorganic/organic matrix modifications, exhibit a higher degree of conversion and wear resistance due to the presence of a cross-linked polymer network [21,22,23]. Since then, gradual improvements in the organic and inorganic matrix structure of these materials have led to the introduction of pure-silicate-technology-based composites.

Studies were conducted comparing an ormocer-based BFC and a methacrylate-based BFC with respect to microleakage, marginal gap formation, and marginal integrity. While similar microleakage values [24] and marginal integrity [25] were observed among the materials in various studies, there were also studies which revealed less marginal gap formation [26,27] and lower microleakage values [28,29] in teeth restored with an ormocer-based BFC. To the authors’ knowledge, while there are studies available, there is limited information in the literature with respect to the effects of the marginal integrity of BFCs used as a liner and marginal relocation material. 

In this study, which was conducted to assist with the selection of restorative materials for clinical use in marginal elevation, ormocer-based BFCs were compared with methacrylate-based BFCs in terms of microleakage and marginal integrity in deep Class II restorations with different layering strategies. 

The null hypotheses in the study are as follows:

(1) Different types of matrix structures (ormocer vs. methacrylate) have no effect on the microleakage values of restorations;
(2) Different layer thicknesses (2 mm vs. 4 mm) do not affect the microleakage values of restorations.

## 2. Materials and Methods

A total of 60 extracted, non-carious human mandibular molars with similar dimensions were included in the study. A dental loupe (×2.5/450; Carl Zeiss, Jena, Germany) was used for the detection of microscopic crack lines or enamel craze lines on the teeth. The number of samples was determined based on a power analysis. The minimum sample size required to detect a significance difference using this test should be at least 10 in each group (60 in total), considering a type I error (alfa) of 0.05, power (1-beta) of 0.99, and an effect size of 1.623. 

**Figure 1:**
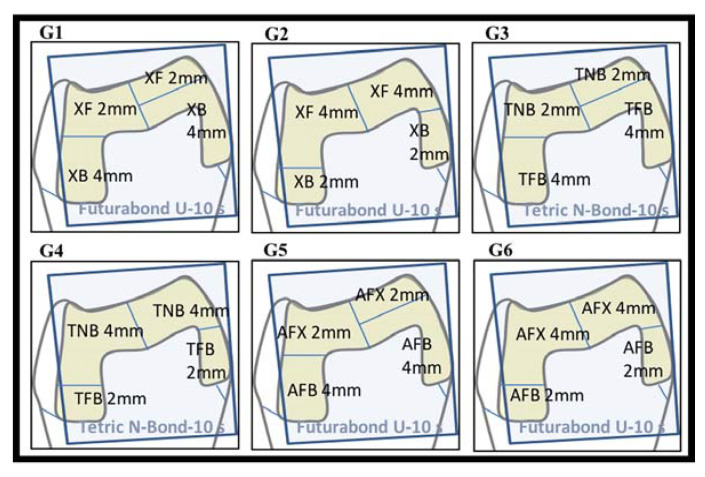
Schematic representation of test groups (Group 1 to 6).

**Figure 2:**
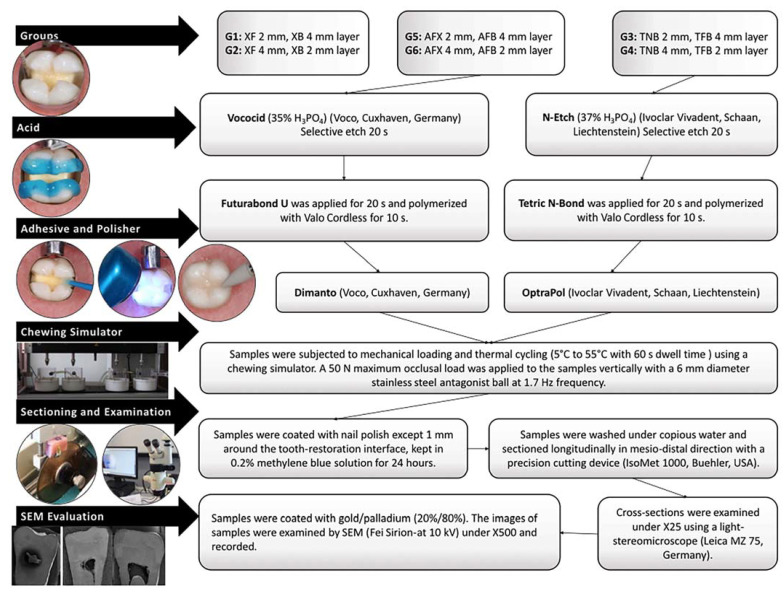
Stages followed for stereomicroscope and SEM evaluation of restorations completed according to manufacturer’s instructions.

The teeth were immersed in an isotonic saline solution containing 0.1% thymol, and all residues were cleaned using an ultrasonic device, followed by fluoride-free paste and a rubber bur. The samples were then embedded in the molds of the chewing simulator using self-cure acrylic (Imicriyl, Konya, Turkey). Mesio-occlusal-distal (MOD) cavities (an occlusal isthmus of 3 mm in width/2 mm in depth and proximal boxes of 4 mm in depth/2 mm in wide) were prepared (as per standard protocol) with cervical margins 1 mm below the cemento-enamel junction (CEJ) on one side and 1 mm above the CEJ on the other. The cavities were prepared by a single researcher (A.A.Ş.) using round and inverted conical burs under water-cooling. A periodontal probe was used for the millimetric measurement of the cavity borders. The prepared teeth were randomly assigned into groups (Figure 1), and the restorations were completed by a single operator (A.A.Ş), in accordance with the manufacturer’s instructions (Table 1; Figure 2). The polymerization distance was standardized by positioning the tip of the curing device at a distance of 6 mm from the cavity floor at the distance allowed by the circumferential matrix band around the tooth. MOD cavities were polymerized from the center of each proximal box, assuming two separate Class II cavities.

In order to mimic the oral environment, thermo-mechanical loading was applied to all samples with a chewing simulator (SD Mechatronik, Feldkirchen-Westerham, Germany) to simulate 1 year of clinical service (240,000 loading cycles), and an immediately tested group was not formed [30] (Figure 2). Thereafter, stereomicroscope (Leica MZ75, Leica Microsystems Pty Ltd, Wetzlar Germany) and SEM (Fei Sirion-10 kV, FEI Company, Hillsboro, OR, USA) evaluations were carried out. Gingival microleakage was recorded using the following scoring system: score 0, no dye penetration; score 1, dye penetration up to half of the cervical wall; score 2, dye penetration up to more than half or the full extent of the cervical wall; and score 3, dye penetration into the cervical and axial walls extending towards the pulp. Coronal microleakage was recorded using the following scoring system: score 0, no dye penetration; score 1, dye penetration into the enamel; score 2, dye penetration beyond the dentinoenamel junction; score 3, dye penetration into the pulpal wall [31].

### Statistical Analysis

All statistical analyses were carried out using the NCSS 2007 software (Number Cruncher Statistical System, Kaysville, UT, USA) program. Descriptive statistical methods were used to evaluate the data, and chi-square tests were used for comparison of qualitative data. A *p*-value < 0.05 was considered statistically significant.

## 3. Results

### 3.1. Microleakage Analysis 

**Figure 3:**
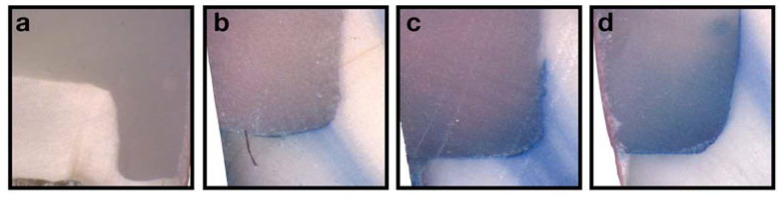
Representation of dye penetration at different scores (×8 and ×25). (**a**) Gingival score 0/Coronal score 0 (×8); (**b**) gingival score 2 (×25); (**c**) gingival score 3 (×25); (**d**) gingival score 3 (×25).

The distribution of the microleakage scores for enamel and dentin margins are shown in Table 2 and Figure 3.

No statistically significant difference was observed in the distribution of coronal and gingival–enamel microleakage values between the XF2 mm + XB4 mm (G1) and XF4 mm + XB2 mm (G2), TNB2 mm + TFB4 mm (G3) and TNB4 mm + TFB2 mm (G4), and AFX2 mm + AFB4 mm (G5) and AFX4 mm + AFB2 mm (G6) groups (*p* = 0.288, *p* = 0.086, and *p* = 0.531, respectively for coronal microleakage; *p* = 0.428, *p* = 0.257, and *p* = 0.113, respectively for gingival–enamel microleakage) (Table 2).

Similarly, no statistically significant difference was found in the distribution of gingival–cementum microleakage values between the mm + XB4 mm (G1) and XF4 mm + XB2 mm (G2), and the AFX2 mm + AFB4 mm (G5) and AFX4 mm + AFB2 mm (G6) groups (*p* = 0.604 and *p* = 0.481, respectively). However, the TNB2 mm + TFB4 mm (G3) group resulted in greater score 3 gingival–cementum microleakage values compared to the TNB4 mm + TFB2 mm (G4) group (*p* = 0.018) (Table 2). 

Considering the coronal microleakage, gingival–enamel microleakage, and gingival–cementum microleakage, no significance was detected between the XF4 mm + XB2 mm (G2), TNB4 mm + TFB2 mm (G4), and AFX4 mm + AFB2 mm (G6) groups (*p* = 0.529, *p* = 0.935, and *p* = 0.529, respectively) (Table 3). A statistically significant difference in coronal microleakage distribution was observed between the XF2 mm + XB4 mm (G1), TNB2 mm + TFB4 mm (G3), and AFX2 mm + AFB4 mm (G5) groups (*p* = 0.048) (Table 3), with the AFX2 mm + AFB4 mm (G5) group exhibiting significantly higher prevalence of score 0 coronal microleakage compared to the TNB2 mm + TFB4 mm (G3) group (*p* = 0.039) (Table 3). However, no significant difference was found between the other groups in terms of coronal microleakage (*p* > 0.05). Moreover, no statistically significant difference was detected in the distribution of gingival–enamel and gingival–cementum microleakage amounts between the XF2 mm + XB4 mm (G1), TNB2 mm + TFB4 mm (G3), and AFX2 mm + AFB4 mm (G5) groups (*p* = 0.112, *p* = 0.563, respectively) (Table 3).

### 3.2. SEM Evaluation

**Figure 4:**
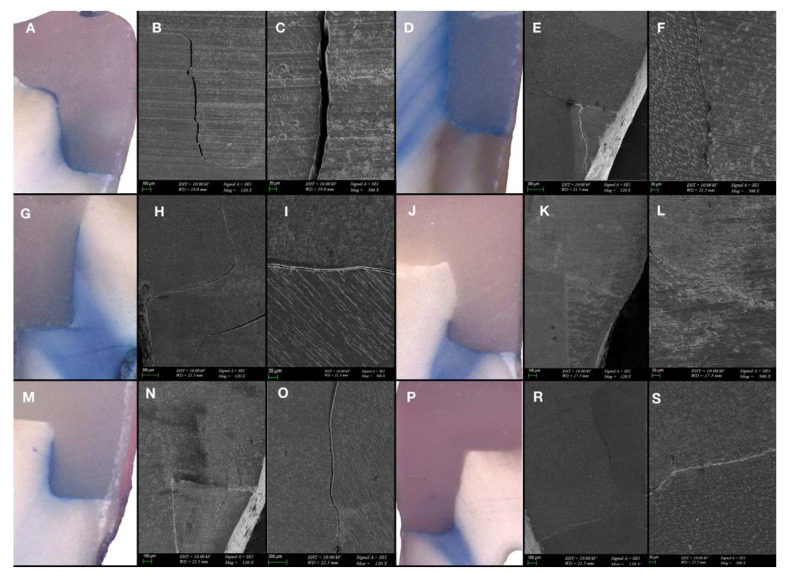
(**A**). Dye penetration of the sample restored with XF2 mm + XB4 mm (G1) (×25); (**B**,**C**) SEM micrograph of the marginal integrity of XF2 mm + XB4 mm (G1) (×120-×500); (**D**) dye penetration of the sample restored with XF4 mm + XB2 mm (G2) (×25); (**E**,**F**) SEM micrograph of the marginal integrity of XF4 mm + XB2 mm (G2) (×120-×500); (**G**) dye penetration of the sample restored with TNB2 mm + TFB4 mm (G3) (×25); (**H**,**I**) SEM micrograph of the marginal integrity of TNB2 mm + TFB4 mm (G3) (×120-×500); (**J**) dye penetration of the sample restored with TNB4 mm + TFB2 mm (G4) (×25); (**K**,**L**) SEM micrograph of the marginal integrity of TNB4 mm + TFB2 mm (G4) (×120-×500); (**M**) dye penetration of the sample restored with AFX2 mm + AFB4 mm (G5) (×25); (**N**,**O**) SEM micrograph of the marginal integrity of AFX2 mm + AFB4 mm (G5) (×120); (**P**) dye penetration of the sample restored with AFX4 mm + AFB2 mm (G6) (×25); (**R**,**S**) SEM micrograph of the marginal integrity of AFX4 mm + AFB2 mm (G6) (×120-×500).

**Figure 5:**
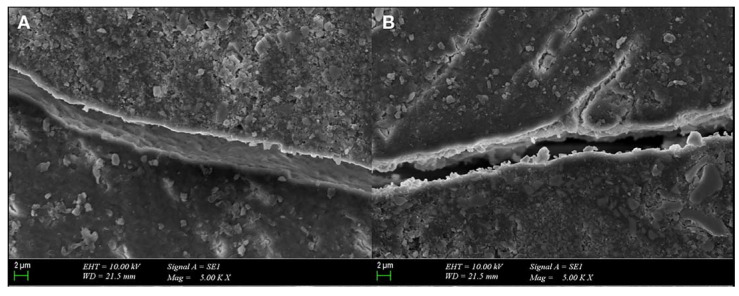
(**A**,**B**) Gap formation in the adhesive is observed in the SEM micrographs (×5000) of the samples from G2 (XF4 mm + XB2 mm) and G3 (TNB2 mm + TFB4 mm).

Samples with a score of 2 or 3 were taken from each group, and SEM micrographs were examined under ×120, ×500, and ×5000 magnifications (Figure 4 and Figure 5). In the G1 (XF2 mm + XB4 mm) and G2 (XF4 mm + XB2 mm) samples with a gingival microleakage score of 3, diffuse dye penetration with discontinuous marginal integrity and gap formations were observed (Figure 4A–C,D,E). In addition, diffuse dye penetration and common gap formation were consistent in the G3 (TNB2 mm + TFB4 mm) sample with a gingival microleakage score of 3 (Figure 4G–I). The dye penetration and gap formation observed in the G4 sample (TNB4 mm + TFB2 mm) with a gingival microleakage score of 3 were consistent (Figure 4J–L). In the G5 sample (AFX2 mm + AFB4 mm) with a gingival microleakage score of 3, dye penetration was not limited to the gingival seat, and progression to the pulpal wall was observed. A common gap formation was observed on the SEM micrographs (Figure 4M–O). Gap formations consistent with dye penetration were observed at the margins of the G6 sample (AFX4 mm + AFB2 mm) which received a microleakage score of 2 (Figure 4P–S).

In the evaluation of SEM micrographs of the samples selected from each group due to their diffuse dye penetration, better marginal integrity was observed in the groups with 2 mm thick flowable BFCs.

## 4. Discussion

Until the mid-1990s, the majority of composites contained greater concentrations of bisphenol A glycidyl dimethacrylate (Bis-GMA) monomer, developed by Rafael Bowen, whereas conventional methacrylate monomers, such as triethylene glycol dimethacrylate (TEGDMA), urethane dimethacrylate (UDMA), and bisphenol A polyethylene glycol diether dimethacrylate (Bis-EMA), were present in smaller quantities [1]. Factors contributing to the failure of methacrylate-based composite restorations include polymerization shrinkage and the resulting stresses [32,33]. Recently, ormocer restoratives based on Ormocer^®^ chemistry were introduced and were supposedly free of bisphenol A or any other methacrylate-based monomers [34]. The aim of the development of ormocer-based composites is to minimize polymerization shrinkage in restorations by modifying the organic matrix and increasing the amount of silicate present in the filler material in these composites.

In the literature, it seems that many different restorative materials are used in deep margin elevation, which is one of the minimally invasive treatment approaches in determining the restoration margin in the treatment of deep Class II cavities [35]. Glass ionomer cements, resin modified glass ionomers, resin composites, flowable composites, and BFCs were used for margin relocation in many studies [17,36]. Nevertheless, there is no consensus among researchers on which material is proper for DME [37]. The fact that the performance of Ormocer-containing composites has not been adequately evaluated in this regard has been one of the important points in the emergence of this study.

Based on the findings of this study, the first null hypothesis (that different types of matrix structures (ormocer vs. methacrylate) would have no effect on the microleakage values of restorations) was partially rejected. The distribution of score 0 coronal microleakage in the G5 (AFX2 mm + AFB4 mm) group was found to be significantly higher than that of the G3 (TNB2 mm + TFB4 mm) group (*p* = 0.039). No statistically significant differences were observed upon comparison of the coronal, gingival–enamel, and gingival–cementum microleakage values in the ormocer (G5 (AFX2 mm + AFB4 mm) and G6 (AFX4 mm + AFB2 mm)) and remaining groups (*p* > 0.05).

Previously, Politi et al. [28] reported results on a comparison of microleakage values after the thermal cycling of ormocer-based composites (Admira Fusion), ormocer-based BFCs (Admira Fusion x-tra), resin-based composites (Tetric EvoCeram), and resin-based BFCs (Tetric EvoCeram BF). The teeth restored with ormocer-based composites and ormocer-based BFCs exhibited lower microleakage scores compared with those restored with resin-based composites and resin-based BFCs. Yarmohamadi et al. [24] reported no statistically significant differences in gingival microleakage values between teeth restored using three different restorative materials (Filtek P60, X-tra fil, and Admira Fusion x- tra), although the number of samples exhibiting no microleakage were higher in the ormocer-based composite group. The current results are consistent with the results of Politi et al. and Yarmohamadi et al. Moreover, in the study conducted by Yarmohamadi et al., Ormocer-based composites were also shown to exhibit lower polymerization shrinkage due to the absence of monomers (such as Bis-GMA, UDMA, 2-hydroxyethyl methacrylate, and TEGDMA) found in conventional composites. Ibrahim et al. [26] compared marginal fit in class II cavities that were restored using four different composite materials (Tetric EvoCeram BF, Filtek BF, Admira Fusion x-tra, and Tetric EvoCeram) and exposed to thermal cycling. In their study, an evaluation of SEM images showed that the ormocer group exhibited the lowest mean values in marginal gap length as well as proximal and cervical gap width. Moreover, teeth restored using Futurabond U and Admira Fusion x-tra had the lowest mean marginal gap length, whereas those restored using Tetric N-Bond Universal and Tetric EvoCeram demonstrated the highest mean marginal gap length. Paganini et al. [27] examined the marginal integrity of different types of composite resin restorations (Tetric EvoCeram BF, Admira Fusion x-tra, Venus BF, SDR, and Filtek Supreme XTE) in Class II cavities of primary molars and found that Admira Fusion x-tra exhibited significantly higher marginal integrity compared with all other groups, both before and after thermo-mechanical loading. The study conducted by Contreras et al. [25] aimed to evaluate marginal adaptation following thermo-mechanical aging in bovine incisors restored with Admira fusion x-tra BF, Tetric N-Ceram BF, and Tetric N-Ceram. The Admira fusion x-tra BF group showed a higher degree of conversion than the Tetric N-Ceram BF group but similar marginal adaptation.

BFCs have translucency [38], varying filler particle content, and contain polymerization modulators in the resin matrix, resulting in an increased curing depth [39]. The increased polymerization depth exhibited by BFCs can be attributed to the higher concentration of UDMA monomer, which provides a greater degree of conversion when compared with Bis-GMA [40]. The transparency and larger filler particle size of x-tra fil and x-tra base composites reduce light scattering and decrease the total surface area of the filler content, thus allowing more photons to penetrate the material [41]. Furthermore, the presence of Ivocerin (Bis-4-(methoxybenzoyl) diethylgermanium Ge-3), an acyl phosphine oxide and dibenzoyl-germanium derivative special photoinitiator system that has a higher absorption spectrum, in combination with camphoroquinone (CQ) and the increased particle size, improve penetration by reducing light scattering, thus contributing to the increase in polymerization depth in Tetric N-Ceram BFC [42,43].

Prior to the formation of the elastic network structure, which indicates that the polymerization reaction has reached the gel point, the polymerization shrinkage produces stresses that are partially compensated by the free flow of the material [44,45]. The rate of polymerization shrinkage influences the stresses produced, with higher rates resulting in greater shrinkage stresses [46]. Controlled polymerization shrinkage may be achieved through the maintenance of the low maximum shrinkage strain rate (Rmax) values and a longer gel time. In a low-viscosity matrix, the concentration of free radicals and monomer activity can be increased, and the time to reach the slowdown stage can be extended. Ivocerin, the photoinitiator in Tetric N-Ceram BF and Tetric N-Flow BF, offers better photopolymerization reactivity than CQ and consequently higher Rmax values [47]. Previous studies have shown that UDMA, TEGDMA, and Bis-EMA exhibited higher Rmax values compared with Bis-GMA due to their low viscosity, whereas the Tetric N-Ceram BFC exhibited an increased Rmax value because of the high Bis-EMA content in its matrix [48,49].

In the current study, methacrylate-based restorations did not show significant differences in microleakage values despite different photoinitiator systems and filler properties.

Hoseinifar et al. [40] restored Class II cavities that extended 1 mm below the CEJ using Tetric N-Ceram BF, x-tra fil, and Tetric N-Ceram. Consistent with the results of the present study, no significant differences in microleakage values were observed between the three composite materials after thermo-mechanical loading, and an examination of SEM images also showed a similar gap formation in all the groups.

The current study aimed to examine the effect of BFC layer thickness on microleakage and marginal integrity; this was achieved by comparing the microleakage between sample subgroups in which low-viscosity and high-viscosity BFCs were applied in layers with thicknesses of 2 mm and 4 mm. The second hypothesis ( that the thickness of the layers (2 mm vs. 4 mm) during material application would not affect the microleakage values of restorations) was also partially rejected as a significant difference in microleakage values was observed between the subgroups of the TNB–TFB composite groups. The distribution of score 3 gingival–cementum microleakage was found to be higher in the G3 group (TNB2 mm–TFB4 mm) compared to G4 group (TNB4 mm−TFB2 mm) (*p* = 0.018).

In the present laboratory study, SEM evaluation showed that flowable composites exhibit significantly reduced marginal continuity and greater interface degradation when applied in increasing thicknesses. The lower filler ratio of flowable composites necessitates use with caution in high-stress-bearing areas. Although the elastic modulus of the flowable composite enables it to act as a stress absorber, in internal stress areas exposed to functional loads, its high monomer content can reduce its mechanical resistance. However, it can be assumed that large amounts of unpolymerized monomers may cause deformations with increasing layer thicknesses [20]. In addition, it should be considered that the curing mode and polymerization duration are also important factors in providing superior mechanical properties of the composite resin [50].

This study had several limitations. First, the data obtained were largely influenced by the experience of the physician as well as the working conditions. In addition, since a chewing simulator and stainless steel ball were used as opposing teeth to simulate the oral environment, it was not possible to imitate the clinical situation exactly.

## 5. Conclusions

Despite the limitations of this study, the following conclusions can be drawn:

1. The matrix structure of BFC restorations in MOD cavities is unable to completely eliminate microleakage under the CEJ;

2. The application of Tetric N-Flow BFC at a thickness of 4 mm results in greater microleakage than its application at a thickness of 2 mm.

As a result, based on the findings of this study, the application of BFCs at a thickness of 2 mm during the restoration of MOD cavities may be preferential in order to minimize microleakage. However, further clinical and laboratory investigations are required to obtain more clinically precise results.

## Figures and Tables

**Table 1:** Materials and equipment used in restorations.

Material and Manufacturer	Monomer and Filler Composition	
X-tra fil VOCO, Cuxhaven, Germany	Bis-GMA, TEGDMA, UDMA, barium aluminum silicate, fumed silica, and pigments Filler Content (wt%): 86	
X-tra base VOCO, Cuxhaven, Germany	Bis-EMA, aluminum, and barium silicate Filler Content (wt%): 75	
Tetric N-Ceram Bulk Fill Ivoclar Vivadent, Schaan, Liechtenstein	Bis-GMA, UDMA, barium glass, prepolymer, ytterbium trifluoride, and mixed oxide Filler Content (wt%): 75–77	
Tetric N-Flow Bulk Fill Ivoclar Vivadent, Schaan, Liechtenstein	Bis-GMA, UDMA, TEGDMA, barium glass, ytterbium trifluoride, and copolymers Filler Content (wt%): 68.2	
Admira Fusion x-tra VOCO, Cuxhaven, Germany	Ormocer matrix, silicon dioxide, and glass ceramics Filler Content (wt%): 84	
Admira Fusion x-base VOCO, Cuxhaven, Germany	Ormocer matrix, silicon dioxide, and glass ceramics Filler Content (wt%): 72	
Futurabond U Universal VOCO, Cuxhaven, Germany	HEMA, Bis-GMA, HEDMA, acidic adhesive monomer, UDMA, catalyst, silica nanoparticle, and ethanol	
Tetric N-Bond Universal Ivoclar Vivadent, Schaan, Liechtenstein	Phosphoric acid acrylate, HEMA, Bis-GMA, UDMA, MDP, MCAP, D3MA, and ethanol	
**Light device**	**Wavelength**	**Light intensity**
Valo Cordless (Ultradent, ABD) 3rd Generation	395–480 nm	Standard mode: 1000 mW/cm^2^

Abbreviations: BIS-EMA: Bisphenol A polyethylene glycol diether dimethacrylate; BIS-GMA: Bisphenol A dimethacrylate; D3MA: Decanediol dimethacrylate; HEDMA: hexamethylene dimethacrylate; HEMA: 2-hydroxyethyl methacrylate; MCAP: Methacrylated carboxylic acid polymer; MDP: Methacryloyloxydecyl dihydrogen phosphate; TEGDMA: Triethylene glycol dimethacrylate; UDMA: Urethane dimethacrylate.

**Table 2:** Evaluation of microleakage scores according to groups.

**Coronal Microleakage**									
**Groups**	**Score**	**BFC-4 mm + fBFC-2 mm** **N(%)**			**BFC-2 mm + fBFC-4 mm** **N(%)**			*p*	
XF + XB (G2-G1)	0	7(70)			5(55.56)			0.288	
	1	3(30)			2(22.22)				
	2	0(0)			2(22.22)				
AFX + AFB (G6-G5)	0	9(90)			8(80)			0.531	
	1	1(10)			2(20)				
TNB + TFB * (G4-G3)	0	6(75)			3(33.33)			0.086	
	1	2(25)			6(66.67)				
**Gingival microleakage**									
**Gingival–Enamel microleakage**					**Gingival–Cementum microleakage**				
**Groups**	**Score**	**BFC-4 mm + ** **fBFC-2 mmN(%)**	**BFC-2 mm +** **fBFC-4 mm N(%)**	***p***	**Groups**	**Score**	**BFC-4 mm +** **fBFC-2 mm N(%)**	**BFC-2 mm +** **fBFC-4 mm N(%)**	***p***
XF + XB (G2-G1)	0	7(70)	4(44.44)	0.428	XF + XB (G2-G1)	0	5(50)	3(33.33)	0.604
	1	1(10)	1(11.11)			1	1(10)	0(0)	
	2	2(20)	2(22.22)			2	2(20)	3(33.33)	
	3	0(0)	2(22.22)			3	2(20)	3(33.33)	
AFX + AFB (G6-G5)	0	8(80)	5(50)	0.113	AFX + AFB (G6-G5)	0	4(40)	3(30)	0.481
	1	1(10)	0(0)			1	2(20)	1(10)	
	2	1(10)	5(50)			2	4(40)	4(40)	
	3	0(0)	0(0)			3	0(0)	2(20)	
TNB + TFB (G4-G3)	0	5(62.50)	2(22.22)	0.257	TNB + TFB (G4-G3)	0	3(37.50)	0(0)	0.018

* Two samples of Tetric N-Ceram Bulk Fill/TNB + Tetric N-Flow Bulk Fill group were excluded from the study due to damage during sectioning with precision cutting device. Abbreviations: AFB: Admira Fusion x-base; AFX: Admira Fusion x-tra; BFC: bulk-fill composite; fBFC: flowable bulk-fill composite; TFB: Tetric N-Flow Bulk Fill; TNB: Tetric N-Ceram Bulk Fill; XB: x-tra base; XF: x-tra fil.

**Table 3:** Comparison of microleakage values in groups in which flowable composites were applied in 2 mm and 4 mm thicknesses.

	Coronal Microleakage		Gingival–Enamel Microleakage		Gingival–Cementum Microleakage	
	BFC-2 mm + fBFC-4 mm	BFC-4 mm + fBFC-2 mm	BFC-2 mm + fBFC-4 mm	BFC-4 mm + fBFC-2 mm	BFC-2 mm + fBFC-4 mm	BFC-4 mm + fBFC-2 mm
XF + XB/AFX + AFB/TNB + TFB	0.048	0.529	0.112	0.935	0.563	0.529
XF + XB/AFX + AFB	0.266	0.582	0.225	0.818	0.731	0.374
XF + XB/TNB + TFB	0.105	0.998	0.423	0.945	0.232	0.416
AFX + AFB/TNB + TFB	0.039	0.558	0.058	0.666	0.305	0.968

Abbreviations: AFB: Admira Fusion x-base; AFX: Admira Fusion x-tra; BFC: bulk-fill composite; fBFC: flowable bulk-fill composite; TFB: Tetric N-Flow Bulk Fill; TNB: Tetric N-Ceram Bulk Fill; XB: x-tra base; XF: x-tra fil.

## Data Availability

The data presented in this study are available upon request from the corresponding author.
